# The Effect of Coronavirus Exposure on CEO Perceptions of Climate Change

**DOI:** 10.3389/fpsyg.2022.943952

**Published:** 2022-06-29

**Authors:** Jin Zhang, Yong Liu

**Affiliations:** ^1^Department of Ideology and Political Education, Maxism School, Wuhan University of Science and Technology, Wuhan, China; ^2^Department of Political Economics, Wuhan University of Science and Technology, Wuhan, China

**Keywords:** COVID-19 pandemic, CEO climate risk perception, pro-environmental behaviour (PEB), global crisis, self-efficacy

## Abstract

CEOs’ personal experiences can influence their perceptions of climate change and their firms’ pro-environmental behavior; a concept termed the experience-perception link. Thus, the experience of the recent COVID-19 pandemic may have caused a change in CEOs’ perceptions of another global threat—climate change. We test this hypothesis by comparing survey measures of climate risk perceptions, self-efficacy, and pro-environmental behaviors among 605 randomly selected CEOs in Wuhan across three phases—(1) before, (2) after the COVID-19 outbreak in Wuhan, and (3) after the COVID-19 had been primarily controlled in Wuhan but was declared a pandemic by the WHO. Harnessing between- and within-subjects variation in COVID-19 exposure, we find a substantial increase in climate change beliefs and actions after the COVID-19 evolved from an epidemic to a pandemic, regardless of subjects’ exposure to the pandemic. We also demonstrate that this change is due to the salience of the global crisis and the feeling of hope elicited by observing effective responses to the crisis, rather than personal experiences solely made from a local health crisis. Our results reveal unexpectedly positive side effects of the abrupt shifts in CEOs’ beliefs and their firms’ pro-environmental behaviors in response to the COVID-19 pandemic.

## Introduction

The outbreak of the COVID-19 pandemic has threatened not only human lives but also the global economy, highlighting the potential consequences of global crises. The pandemic may also have given individuals pause for thought about how the world can work together to address a global emergency more effectively. Although climate change and COVID-19 are two different challenges, they share many key elements, e.g., the global nature of a threat, public health concerns, changes in living standards and social norms, and significant consequences for future generations. These similarities lead us to question whether the COVID-19 pandemic has reduced “psychological distance” and shifted public beliefs and actions toward another global threat—climate change. This study aims to empirically examine whether CEOs’ personal experiences, such as the COVID-19 pandemic, can influence their perceptions of climate change and their firms’ pro-environmental behavior. We use a large-scale three-wave survey to answer this question by comparing climate risk perceptions, self-efficacy (beliefs in one’s capacity to affect change), and pro-environmental behaviors among 605 CEOs from 605 randomly selected SMEs (Small and medium-sized enterprises) in Wuhan, China^1^.

Our survey spans three critical phases of the COVID-19 crisis^2^ : before the first case of COVID-19 was identified (October 2019), shortly after the initial COVID-19 outbreak in Wuhan (March 2020), and after COVID-19 was elevated to pandemic status but new infections in Wuhan had slowed, and the city had begun to reopen (April 2020). This time variation allows us to (1) identify the within-subject effect of COVID-19 by comparing survey responses before and after the outbreak of COVID-19, and (2) measure effect heterogeneity as COVID-19 evolved from an epidemic to a pandemic, which allows us to differentiate between a local and a global health threat. Our sample consists of 605 CEOs in Wuhan, 569 of whom returned to their hometowns before survey Waves 2 and 3 for semester breaks and the Chinese Lunar New Year celebrations^3^. This provides quasi-random variation in exposure to the pandemic, which allows us to study the between-subject effect of exposure to COVID-19 based on the geographical variation.

We focus on three measures to examine the impact of COVID-19 on climate change activism. Two measurements capture climate change beliefs—climate risk perceptions and self-efficacy. The third assesses individuals’ willingness to act in a way that addresses climate change—pro-environmental behaviors. We find that fear about a future global crisis plays a prominent role in changing subjects’ climate risk perceptions. The change is systemic regardless of their exposure to the pandemic. Previous studies have shown that climate change awareness and risk perception can be influenced through effective affective stimuli and the associated emotional response (e.g., fear, worry and grief) (e.g., Herrnstadt and Muehlegger, 2014; Zaval et al., 2014; McDonald et al., 2015; van der Linden, 2015; Brügger et al., 2016; Lang and Ryder, 2016; Geiger et al., 2017; Curnock et al., 2019; Bu et al., 2021; Tian et al., 2022). However, elicited negative emotions such as fear may cause individuals to distance themselves or disengage and may negatively influence their beliefs in their ability to address climate change, a phenomenon termed the self-efficacy barrier (e.g., O’Neill et al., 2013; Barnett et al., 2016; Metag et al., 2016; Xue et al., 2020, 2021; Bu and Liao, 2021; Wan et al., 2021). Thus, affective experience-induced stimuli would increase not only the salience of the issue of climate change but also the sense of being able to do something—but few motivations, if any, seem to do both. Our Wave 3 survey was administered when the COVID-19 had been effectively controlled in Wuhan. This offers a unique setting for us to test whether observing an effective response to the crisis can elicit an emotion of hope and consequently help individuals overcome the self-efficacy barrier. We find supportive evidence that subjects’ self-efficacy increased after COVID-19 was under control in Wuhan. We also test whether the increased beliefs in climate change risk and self-efficacy translated into pro-environmental actions. Previous studies have documented a moderate relationship between climate change attitudes and pro-environmental behavior (e.g., Hines et al., 1987; Bamberg and Möser, 2007; O’Neill and Nicholson-Cole, 2009; Xie et al., 2019; Bu et al., 2020). Our findings contribute to the literature by showing that the COVID-19 pandemic has shifted not only individuals’ beliefs in climate change risk but also their actions toward addressing it.

Our results offer an essential contribution to a large body of literature on raising CEOs’ concern about and engagement with climate change. While our study is the first to examine how the worldwide COVID-19 pandemic has affected CEOs’ climate change activism, we also provide novel findings showing that changes in climate change beliefs and actions are more linked to the salience of a global threat than to the belief that climate change was a cause of the COVID-19 pandemic. In general, our results contribute to the literature by documenting the impact of individual-level global crisis experiences on subsequent beliefs about and corporate actions toward climate change. Additionally, our results contribute to the literature that exploits the positive side of COVID-19 on human thoughts and behaviors.

## Methodology

### Sample Selection

Our sample consisted of 605 randomly selected CEOs in Wuhan. In this study, we partner with a Wuhan-based survey firm. The survey company initially sent invitations to 1,400 SMEs in Wuhan with more than five employees at random, and 605 of them consented to take part in the survey. 76 of the 605 SMEs in our sample are in the real estate sector, 35 in the factory sector, 48 in the construction sector, 102 in the tourism & hospitality sector, 53 in the vehicle services sector, 29 in the trade services sector, 72 in the personal service sector, 82 in the general services sector, 66 in the processing sector, 18 in the agricultural products sector, and 24 in the transportation sector.

Wuhan was ground zero of the COVID-19 outbreak and undoubtedly one of the most impacted places; the majority of infected cases in China were located in Wuhan. In Wave 1 of our survey, all subjects were located in the city of Wuhan. Then, winter break for the semester at WUST started on January 11, and most CEOs from other provinces were able to return to their homes as planned for the Chinese Lunar New Year celebrations. As the province of Hubei became quarantined and effectively locked down shortly thereafter, CEOs from other provinces were denied to return to Wuhan. Therefore, in Waves 2 and 3, 94% of the subjects (569) were located in cities outside of Wuhan in parts of China with substantially lower exposure to COVID-19. The surveys were administered by a survey company called Wenjuanxing in China.

### Experimental Design

We conducted a three-wave survey between October 2019 and April 2020. The first wave was from October 16, 2019, to October 18, 2019^4^. The survey consisted of several parts. First, CEOs’ provided demographic information such as their age, sex, date of birth, and birth province. We then asked nine questions about climate risk perceptions following O’Connor et al. ^17^ and Leiserowitz^18^, 1 question on self-efficacy following Metag et al.^10^, and 9 questions on pro-environmental behavioral intentions following Bernauer and McGrath^19^. The detailed survey questions are provided in the survey design section.

The second survey wave was conducted from February 28, 2020, to March 3, 2020.

By then, the city of Wuhan had entered lockdown (on January 23, 2020), and most CEOs in our sample from regions outside of Wuhan had left the city for the lunar new year holiday. We thus administered an online survey to the same subjects. The online survey tool allowed us to capture precise information about subjects’ locations. We mapped the provided geolocation coordinates to cities and provinces across China. In addition to the questions from our baseline survey, we included one question assessing fear due to exposure to the virus and one question about why subjects thought COVID-19 could be linked with climate change. Details on these questions are provided in the survey design section.

The third wave of the survey was conducted from April 15, 2020, to April 22, 2020. On April 8, 2020, lockdown measures were set to be eased in Wuhan. The restrictions were eased following a reduction in the number of daily reported infected cases, with reports suggesting that Wuhan had had only two new confirmed cases in the previous two weeks. People were permitted to leave the city of Wuhan for the first time since the lockdown was imposed on Monday, January 27. Passenger trains began to depart the city, and highways were opened to outbound traffic. While travel restrictions have since eased even further in Wuhan, strict control measures continue nationwide, and residents are still being encouraged to remain within their neighborhoods and avoid travel outside of the city unless it is essential. Our sample of subjects remained in their hometown cities after the Wave 2 survey. We administered one more online follow-up survey round to the same subject pool as in our first and second wave surveys. In addition to the questions from our second wave survey, we included one more question about subjects’ attitudes toward the lockdown measures, such as quarantine and social distancing, that were taken during the outbreak of the virus. Details on the question are provided in the survey design section.

### Survey Design

For all questions listed below, we asked subjects to indicate their level of agreement/disagreement on a 5-point scale (1 = very strongly disagree; 5 = very strongly agree). We measured subjects’ fear of COVID-19 in 1 question: (1) “Are you afraid of COVID-19?” To measure subjects’ climate risk perceptions, we asked 1 question about their general concerns about climate change: (2) “How concerned are you about climate change?” We asked seven questions about their perceptions of the threat of climate change during the next ten years as follows: (3) “Global warming is already a global threat,” (4) “The world is seeing an increasing rate of environmental damage,” (5) “People’s living standards on earth will decrease,” (6) “Worldwide water shortages will occur,” (7) “Worldwide, we have seen increased rates of serious diseases,” (8) “My standard of living will decrease,” and (9) “My chances of getting a serious illness will increase.” To measure subjects’ beliefs in their ability to affect climate change (self-efficacy), we asked 1 question: (10) “I feel that I can do something about climate change. To measure subjects” pro-environmental behavioral intentions, we asked eight questions: (11) “I keep the pressure/flow of the shower at a rate lower than what I consider to be ideal for saving water,” (12) ‘I limit the time I spend in the shower to reduce my water consumption, (13) “I set the air conditioner temperature relatively high in summer to save energy,” (14) “When I boil water, I only boil as much as I need,” (15) “I switch appliances off instead of leaving them on standby,” (16) “I turn off the shower when I soap myself down,” (17) “I switch off the appliances when they are not in use,” and (18) “I always sort the waste.” To measure subjects’ opinions about the link between the COVID-19 crisis and climate change, we asked two questions: (19) “The COVID-19 outbreak was caused by damage to the natural environment” and (20) “Climate change is as serious a global crisis as COVID-19 is.” To measure individual social responsibility, we asked one question: (21) “Quarantines and social distancing are effective measures to prevent the spread of COVID-19.”

### Regression Analysis

Our OLS regression analysis aimed to answer two questions: (1) How has COVID-19 impacted subjects’ attitudes toward climate change, and how has that attitude evolved over different phases of the COVID-19 crisis? (2) What is the mechanism through which COVID-19 has influenced subjects’ attitudes toward climate change? The dependent variables included (1) *climate risk perceptions*, which is the aggregate mean score of responses to the nine questions about subjects’ climate risk perceptions; (2) *self-efficacy*, which is the score of responses to one question measuring subjects’ beliefs in their ability to affect climate change; and (3) *pro-environmental behavioral intentions*, measured by the aggregate mean score of responses to the eight questions measuring subjects’ pro-environmental actions. The explanatory variables were *Wuhan subjects*, an indicator variable taking the value of one if subjects were quarantined or stayed in Wuhan city during the Wave 2 and 3 surveys. *Wave two* and *Wave three* indicate March 2020, the time of our second survey wave, and April 2020, showing our third survey wave, respectively. The two survey wave variables and their interaction with the Wuhan location variable were the variables of interest. Robust standard errors were used.

## Empirical Findings

### Emotional Responses to COVID-19

**FIGURE 1:**
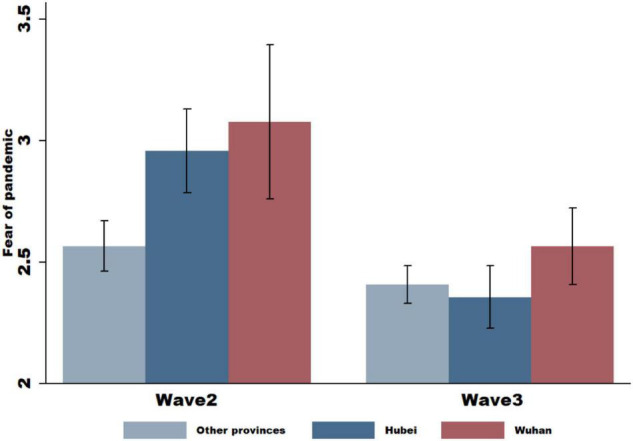
Subjects’ self-reported fear of COVID-19. In the following figure, we plot subjects’ self-reported fear of COVID-19. We plot the responses by subjects who were quarantined in Wuhan, subjects who were quarantined in the province of Hubei (but outside of Wuhan), and subjects in different provinces in China. In *Wave 2*, we plot the mean values of responses to the question asking whether subjects were afraid of the COVID-19 pandemic during the COVID-19 outbreak in Wuhan when the city was under lockdown (our Wave 2 survey). In *Wave 3*, we plot the mean values of responses to the question asking whether subjects were afraid of COVID-19 when it was declared a pandemic and the Wuhan city lockdowns were eased (our Wave 3 survey). Responses were given on a scale between (1) and (5), corresponding to “not afraid at all” to “very afraid,” respectively, and 95% confidence intervals are displayed.

Emotional responses to an experienced threat can influence climate change awareness and beliefs. Thus, we tested whether the pandemic has evoked a feeling of fear using the question “Are you afraid of COVID-19?” Responses ranged from 1 to 5, corresponding to “low” and “high” levels of fear. We find that after the outbreak of COVID-19 in Wuhan (Figure 1, Wave 2), subjects located in the city of Wuhan or in Hubei Province, where the crisis had been much more severe than in other places in China, reported a higher level of fear about COVID-19 in general. This suggests a significant increase in fear in response to COVID-19, particularly for those with greater exposure to the crisis. When the COVID-19 virus evolved into a worldwide pandemic, and the focus shifted away from Wuhan (quarantine conditions in China were eased) (Figure 1, Wave 3), subjects reported a lower level of fear about COVID-19 regardless of their exposure to the pandemic. This suggests that the effective control of the virus in its place of origin may have improved the confidence of the public and provided relief. These results are further supported by the summary statistics reported in Table 1, where the mean score of responses to the question decreased from 2.704 (Wave 2) to 2.403 (Wave 3), and the difference was significant at the 5% level (*p-value* = 0.000).

**TABLE 1:** Summary statistics.

	Wave 1: mean (± s.e.m.)	Wave 2: mean (± s.e.m.)	Wave 3: mean (± s.e.m.)	Significance of changes from wave 1 to wave 2 (two-tailed)	Significance of changes from wave 1 to wave 3 (two-tailed)	Significance of changes from wave 2 to wave 3 (two-tailed)
**Fear of COVID-19** (on a 5-point scale: 1 = lowest, 5 = highest)						
‘Are you afraid of COVID-19?’		2.704 (1.096)	2.403 (0.791)			↓P = 0.000
**Attitude towards climate change** (on a 5-point scale: 1 = lowest, 5 = highest)						
(1) **Perceived climate risk** Average score of the 9 perceived climate change risk questions	3.571 (0.028)	3.633 (0.026)	4.031 (0.025)	↑ P = 0.054	↑ P = 0.000	
(2) **Pro-environmental behaviors** Average score of the 8 pro-environmental behavior questions	3.891 (0.026)	3.909 (0.0229)	4.111 (0.023)	↑ P = 0.308	↑ P = 0.000	
(3) **Self-efficacy** “I feel I can do something about climate change.”	3.696 (0.039)	3.618 (0.042)	4.221 (0.035)	↓P = 0.909	↑P = 0.000	
**Mechanism** (on a 5-point scale: 1 = lowest, 5 = highest)						
(1) “The outbreak of COVID-19 was caused by damage to the natural environment.”		3.390 (0.038)	3.238 (0.038)			↓P = 0.523
(2) “Climate change is as serious a global crisis as COVID-19 is.”			3.967 (0.033)			

*This table reports the summary statistics (mean and standard deviation in brackets) of subjects’ responses to the survey questions. Part (a) reports the responses to 1 question about fear of COVID-19; part (b) shows the responses regarding attitudes toward climate change, including perceived climate change risk, self-efficacy, and pro-environmental behavior; and part (c) presents the responses relating to the perceived link between COVID-19 and climate change. The p-value shows the significance of the repeated-measures t-test (or paired two-sample t-test).*

### Climate Change Beliefs and Actions

**FIGURE 2:**
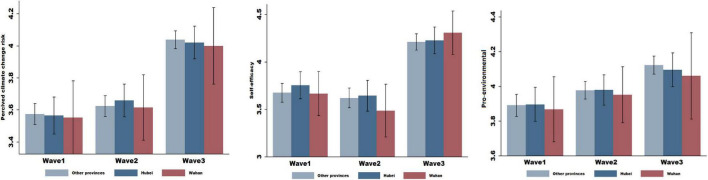
Subjects’ climate change beliefs and actions. In the following figure, we plot subjects’ climate change beliefs and actions, including their perceived climate change risk, self-efficacy (belief in their ability to affect change), and pro-environmental behaviors. We plot responses by subjects who were quarantined in Wuhan, subjects who were quarantined in the province of Hubei (but outside of Wuhan), and subjects in different provinces in China. In *Wave 1*, we plot the mean values of responses to 9 questions about subjects’ climate risk perceptions (perceived climate change risk), 1 question about subjects’ belief in their capacity to address climate change (self-efficacy), and 8 questions about subjects’ willingness to take action to address climate change (pro-environmental behaviors) before the outbreak of COVID-19. In *Wave 2*, we plot the mean values of responses to these questions after the COVID-19 outbreak in Wuhan, when the city was under lockdown. In *Wave 3*, we plot the mean values of responses to these questions when COVID-19 was declared a pandemic, but the Wuhan city lockdowns were set to be eased. Responses were given on a scale between (1) and (5), corresponding to “strongly disagree” and “strongly agree,” respectively, and 95% confidence intervals are displayed.

We next examine whether the elicited fear influenced subjects’ climate change beliefs and actions, including their climate risk perceptions, beliefs in their ability to do something about climate change (self-efficacy), and willingness to act to address climate change (pro-environmental behaviors). We measured climate risk perceptions using a single question that relates to the general concerns about climate change risk, “In general, how concerned are you about global climate change?,” and eight questions following O’Connor et al. (1999) and Leiserowitz (2006) (the detailed questions are provided in *Methods*). Responses to the questions were given on a scale of 1 to 5, corresponding to “strongly disagree” and “strongly agree.” We averaged the answers of 9 questions into an equally weighted scale ranging from 1 to 5, representing “low” and “high” climate risk perceptions, respectively. Compared to their responses from pre-outbreak times (Wave 1, left panel of Figure 2.), subjects’ beliefs in climate risk increased significantly after COVID-19 was elevated to pandemic status (Wave 3, left panel of Figure 2.) but did not change after the initial outbreak of COVID-19 in Wuhan (Wave 2, left panel of Figure 2). Table 1 reports the average score of the nine questions, showing that the average score increased slightly, from 3.571 (Wave 1) to 3.898 in Wave 2 and jumped to 4.031 in Wave 3. The increase from Wave 1 to Wave 3 amounted to 0.46, which was statistically significant at the 5% level (*p-value* = 0.000) and economically substantial given the mean value of 3.571 in Wave 1. Multiple regression analysis further confirmed these results (Table 2), where the dependent variable was the average score of the nine questions, and *Wave 2*, *Wave 3*, and *Wuhan* were independent variables indicating the responses of subjects from Wave 2 and 3 surveys and the reactions of the subjects who were quarantined in Wuhan during the two surveys. We controlled for subjects’ demographic characteristics, such as age and gender. The coefficient of *Wave 3* was significant (i.e., coefficient = 0.462, *s.d.* = 0.036), but the interaction term Wave 3*Wuhan was insignificant (i.e., coefficient = –0.009, *s.d.* = 0.091), indicating that climate risk perceptions were positively shifted after the COVID-19 had spread globally, regardless of subjects’ individual exposure to the virus.

**TABLE 2:** Regression results.

	(1) Climate risk perceptions	(2) Self-efficacy	(3) Pro-environmental behaviors
Wave two	0.051	0.026	−0.066
	(0.041)	(0.032)	(0.065)
Wave three	0.462***	0.228***	0.545***
	(0.036)	(0.042)	(0.059)
Wuhan subjects	−0.009	0.007	0.079
	(0.067)	(0.059)	(0.086)
Wave two*Wuhan subjects	0.050	−0.036	−0.046
	(0.092)	(0.078)	(0.126)
Wave three*Wuhan subjects	−0.009	−0.020	−0.070
	(0.091)	(0.084)	(0.120)
Control variables	Yes	Yes	Yes
R2	0.095	0.029	0.073
Observations	1,812	1,812	1,812

This table reports the regression analysis results. The dependent variable in Column (1) is climate risk perceptions, which is the aggregate mean score of responses to the 8 questions in part (b) of the survey. Column (2) is self-efficacy, which is the score of part (c) in the survey, and Column (3) is pro-environmental behaviors, measured by the aggregate mean score of responses to the 8 questions in part (d) of the survey. The explanatory variables are Wuhan subjects, an indictor variable that takes the value of one if the subject was quarantined or stayed in the city of Wuhan during the period of the Waves 2 and 3 surveys. Wave two and Wave three indicate March 2020 for our second survey wave and April 2020 for our third survey wave, respectively. The variables of interest are the two survey wave variables and their interaction with the Wuhan location variable. The robust standard errors used are reported in brackets. *** indicate significance at the 1% level, respectively.

We next investigated subjects’ self-efficacy, i.e., their sense of being able to do something about climate change. Following Metag et al. (2016), we measured self-efficacy using the question, ‘I feel that I can do something about climate change. The answers ranged from 1 to 5, corresponding to “strongly disagree” and “strongly agree,” respectively. Compared with responses from normal times (Wave 1, middle panel of Figure 2), subjects, especially those who were quarantined in Wuhan, reported a slightly lower level of self-efficacy after the outbreak of COVID-19 in Wuhan (Wave 2, middle panel of Figure 2). However, these changes were insignificant (*p-value* = 0.909). A possible explanation is that witnessing the outbreak of a health crisis without seeing effective actions to address it may decrease people’s sense of being able to do something about another threat, e.g., climate change. However, the indirect link between COVID-19 and climate change may have moderated the effect. In contrast, we observed that subjects’ self-efficacy increased considerably after COVID-19 had spread globally. Still, the quarantine restrictions in Wuhan were eased, marking a milestone in gaining control over the virus (Wave 3, middle panel of Figure 2). These findings were confirmed by the results shown in Table 1, which show that the mean score of responses to the question increased from 3.696 (Wave 1) to 4.221 (Wave 3); this increase was statistically significant at the 5% level (*p-value* = 0.000). The results from regression analysis (Table 2) provide further evidence, where the coefficient of *Wave 3* was significantly positive (coefficient = 0.228, *s.d.* = 0.042) but that of *Wave 2* was insignificant (coefficient = 0.026, *s.d.* = 0.032). Meanwhile, the interaction term Wave 3*Wuhan was insignificant (coefficient = –0.020, *p-value* = 0.084), suggesting that subjects’ self-efficacy increased after they observed an effective response to the crisis in Wuhan and that this increase was independent of their exposure to the crisis.

Finally, we tested whether subjects’ pro-environmental behaviors shifted in response to COVID-19. Following Bernauer and McGrath (2016), we measured pro-environmental behaviors using eight questions that presented a set of scenarios and asked about the willingness to act in environmentally friendly ways. The answers ranged from 1 to 5, corresponding to “strongly disagree” and “strongly agree,” respectively (detailed questions are provided in *Methods*). We averaged the answers of the eight questions into an equally weighted scale ranging from 1 (low level of pro-environmental behaviors) to 5 (high level of pro-environmental behaviors). We found that after experiencing the local outbreak of COVID-19 in Wuhan (Wave 2, right panel of Figure 2), subjects stated a slightly higher level of willingness to act in pro-environment ways, confirmed by the mean scores reported in Table 1, which increased from 3.891 (Wave 1) to 3.976 (Wave 2). However, the increase was insignificant at the 5% level (*p-value* = 0.308). When the health crisis evolved from the local to the global level, the mean score of answers to the eight questions further increased to 4.111; this increase was statistically and economically significant. The regression analysis further supported these results.

Overall, our results showed a significant increase in climate risk perceptions and self-efficacy, which translated into pro-environmental behaviors, from normal times before the COVID-19 outbreak to the time that it had evolved into a global health crisis. It is plausible that this effect was partially driven by a belief that there is a direct link between the COVID-19 pandemic and damage to the natural environment (potentially as a source of the pandemic), or an increase in fear about an analogous global crisis. Our findings of no significant changes after the local outbreak of COVID-19 in Wuhan can somewhat rule out the first explanation. We will further explore these two potential explanations in the following section.

### Mechanism Analysis: A Next Global Crisis or a Source of the COVID-19

We attempted to disentangle the two aforementioned explanations by analyzing responses to the following two questions: “The COVID-19 outbreak was caused by damage to the natural environment,” which was asked in both Wave 2 and Wave 3, and “Climate change is as serious a global crisis as COVID-19 is,” which was asked only in Wave 3. The responses were given on a scale of 1 to 5, corresponding to “strongly disagree” and “strongly agree,” respectively.

**FIGURE 3:**
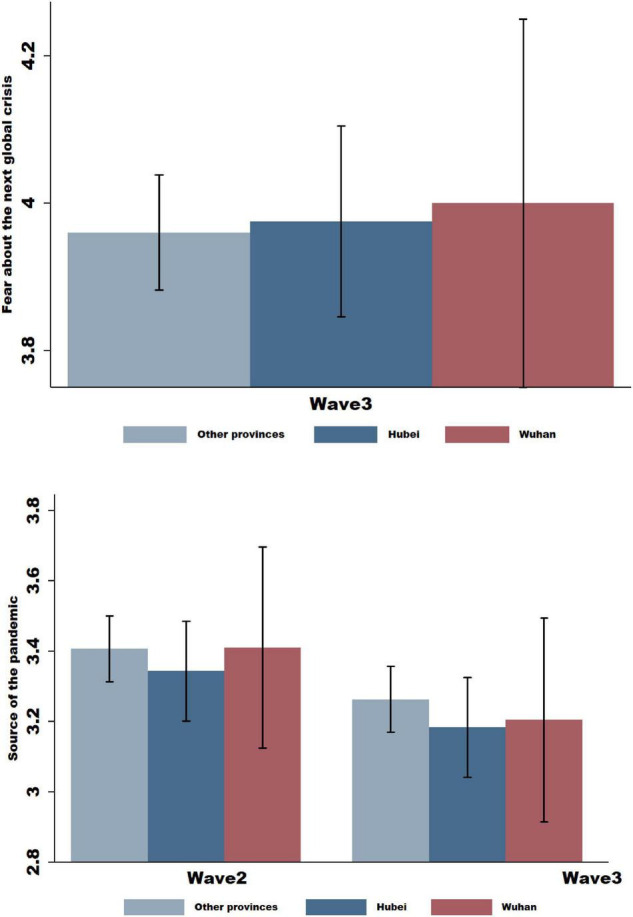
Mechanism analysis: A next global crisis or a source of the COVID-19. In the following figure, we plot subjects’ perceived link between COVID-19 and climate change. We plot the responses by subjects who were quarantined in Wuhan, subjects who were quarantined in the province of Hubei (but outside of Wuhan), and subjects in different provinces in China. In *Wave 2*, we plot the mean values of responses to the item “The COVID-19 outbreak was caused by damage to the natural environment” during the coronavirus outbreak in Wuhan when the city was under lockdown. For *Wave 3*, we plot the mean values of responses to the two items ‘The COVID-19 outbreak was caused by damage to the natural environment’ and “Climate change is as serious a global crisis as COVID-19 is” during the period when COVID-19 was declared a pandemic and the Wuhan city lockdown was being eased. Responses were given on a scale between (1) and (5), corresponding to “strongly disagree” and “strongly agree,” respectively, and 95% confidence intervals are displayed.

We found that fear about a future global crisis played the main role in changing subjects’ climate change beliefs and actions. Subjects were more likely to agree that the COVID-19 pandemic is as much a global crisis as climate change is (Figure 3). While the mean score of the item “Climate change is as serious a global crisis as COVID-19 is” was 3.967, the mean score of the item “The COVID-19 outbreak was caused by damage to the natural environment” was only 3.238 (Table 1). The difference amounted to 0.729, accounting for 14.5% of the 5-point scale. Additionally, the responses to the two questions across subjects who were quarantined in Wuhan, quarantined in Hubei Province (outside of Wuhan), and quarantined in other provinces showed no significant difference, suggesting that subjects’ perceived link between COVID-19 and climate change was not affected by their exposure to the virus.

### Heterogeneity Analysis: Attitudes Toward Social Responsibility

**FIGURE 4:**
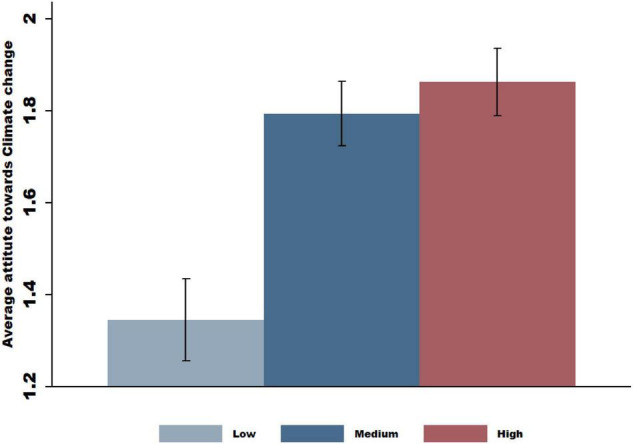
Heterogeneity analysis: attitudes toward social responsibility. In the following figure, we examine whether the impact of COVID-19 on subjects’ attitude toward climate change depended on their level of social responsibility. We measured individual social responsibility using the item “Quarantines and social distancing are effective measures for preventing the spread of COVID-19.” Responses were given on a scale of 1 to 5, corresponding to “strongly disagree” and “strongly agree,” respectively. We sorted our sample of subjects into three cohorts representing subject groups with low, medium and high levels of social responsibility. We then plotted the responses regarding the change in attitude toward climate change (the change in the mean score of responses to the 18 questions related to climate change) from Wave 2 to Wave 3 according to subjects in the low, medium and high social responsibility groups, and 95% confidence intervals are displayed.

Finally, we examined whether the shifts in climate change beliefs and actions due to COVID-19 varied across individuals. We focused on individuals’ attitudes toward social responsibility and tested the hypothesis that larger shifts would happen among more socially responsible individuals. During the COVID-19 pandemic, most nations have enforced social distancing rules, quarantines, and/or lockdowns to contain the spread of the virus. These mobility restrictions require collective and unified action. We assumed that individuals’ attitudes toward these collective actions might reflect their attitudes toward social responsibility. We thus measured individual social responsibility using responses to the question, “Quarantines and social distancing are effective measures to prevent the spread of COVID-19.” Subjects answered this question on a scale of 1 to 5, corresponding to “strongly disagree” and “strongly agree,” respectively. We sorted our subjects into three groups (representing low to high levels of social responsibility) and examined how shifts in climate change beliefs and actions (measured by the difference between the Waves 1 and 3 survey responses) varied across these groups. We aggregated the answers to all of the aforementioned questions into equally weighted answers from 1 to 5. We found that the positive shift in climate change beliefs and actions monotonically increased from the low (mean score = 1.45) to high (mean score = 1.85) social responsibility groups, and the highest changes were observed in the subject group most in favor of quarantines or social distancing measures (Figure 4). Thus, we conclude that the COVID-19 pandemic has caused positive shifts in the public’s climate change beliefs and actions, and the effect is more pronounced among people with high stated levels of social responsibility.

## Limitations and Future Research

The study’s design has several advantages, but it also comes with drawbacks. One limitation of this study is that participants were aware that they were participating in an experiment. This, of course, could impact their behavior, especially their attitude toward their social preference. However, because individuals in different treatment groups received identical questions, we could account for potential effects associated with the concept of monitoring. Although we randomly assigned participants into other treatment groups to account for potential monitoring effects, it would be interesting to observe if comparable results occur when individuals are unaware they are being monitored.

We also propose some future research directions in this study. The role of peer effects in decision making has been largely explored in many contexts, such as green product adoption, saving and borrowing decisions (Georgarakos et al., 2014; Bu et al., 2021). It is well documented in those studies that people can learn from their friends’ or colleagues’ experiences and can be influenced by their choices (Hirshleifer, 2020). While peer impacts are believed to influence individuals’ perceptions of climate change danger and pro-environmental action, little study has been conducted thus far. Additional research should be performed to ascertain whether and how an individual’s enhanced pro-environmental behavior affects peers. Moreover, for future research, machine learning techniques could be used to assess treatment effects in this type of trial.

## Conclusion

Our study examined how CEOs’ climate change beliefs and corporate pro-environmental behavior evolved from before the COVID-19 outbreak to when it had become a global health crisis. We use repeated survey data from a large panel of subjects based in Wuhan, China. Our identification strategy exploited the fact that the COVID-19 outbreak evolved from an epidemic in Wuhan to a global pandemic. This variation allowed us to differentiate between local and global crisis perspectives. The sample size in our study is substantially greater than comparable studies. For example, Brügger et al. (2016) tested whether psychological distance from climate change predicted pro-environmental intentions with 252 subjects in their experiments. Metag et al. (2016) investigated whether expediting the COVID-19 pandemic causes greater risk-taking with only 231 subjects. The relatively larger sample size allows us to estimate the effect with a desired statistical power.

We found that while the CEOs showed an emotional response to COVID-19, as measured by higher levels of fear during the COVID-19 outbreak in Wuhan, this was not a constant determinant affecting their beliefs and corporate behavior toward climate change. However, when COVID-19 became a global pandemic, fear translated into a higher perception of climate risk, a more heightened sense of being able to do something about climate change, and a higher willingness to act to address climate change. We argue that this is mainly explained by subjects’ belief in an analogous future global crisis after they observed the consequences of a global health crisis and felt hope after observing the effective responses to that crisis. At the same time, heterogeneity in exposure to COVID-19 did not differentially affect climate change beliefs and actions; instead, on average, all subjects surveyed showed a large and significant increase.

## Data Availability Statement

The raw data supporting the conclusions of this article will be made available by the authors, without undue reservation.

## Author Contributions

All authors listed have made a substantial, direct, and intellectual contribution to the work, and approved it for publication.

## Conflict of Interest

The authors declare that the research was conducted in the absence of any commercial or financial relationships that could be construed as a potential conflict of interest.

## Publisher’s Note

All claims expressed in this article are solely those of the authors and do not necessarily represent those of their affiliated organizations, or those of the publisher, the editors and the reviewers. Any product that may be evaluated in this article, or claim that may be made by its manufacturer, is not guaranteed or endorsed by the publisher.

## Footnotes

1 The survey was initially set up prior to the COVID-19 outbreak to study individuals’ risk preference, climate risk perceptions, self-efficacy and pro-environmental behaviors. Due to the unique location of the study, we decided to amend the study design once the World Health Organization (WHO) declared COVID-19 as a pandemic (World Health Organization [WHO], 2020).

2 Details of the survey, sample, experiment design and regression analysis methods can be found in the *Methods* appendix.

3 This survey was originally for another project on beliefs about luck and investment behaviors. We also asked questions regarding climate change beliefs and actions.
